# Treatment‐free remission in CML patients with prior TKI failure is not influenced by previous *BCR::ABL1* kinase domain mutations

**DOI:** 10.1111/bjh.70794

**Published:** 2026-09-02

**Authors:** Simone Claudiani, Silvia Metelli, Myrto Katraki, Fiona Fernando, Andrew J. Innes, Chloe Hayden, Dragana Milojkovic

**Affiliations:** ^1^ Centre for Haematology, Department of Immunology and Inflammation Faculty of Medicine, Imperial College London London UK; ^2^ Department of Haematology Hammersmith Hospital, Imperial College Healthcare NHS Trust London UK; ^3^ Centre of Research in Epidemiology and Statistics (CRESS) University of Paris, INSERM, INRA Paris France; ^4^ Assistance Publique ‐ Hôpitaux de Paris (APHP) Paris France; ^5^ North West London Pathology, Hammersmith Hospital Imperial College Healthcare NHS Trust London UK

**Keywords:** CML, KD mutations, TFR, TKI failure

To the Editor,

Treatment‐free remission (TFR) has become an important therapeutic goal in chronic‐phase chronic myeloid leukaemia (CML‐CP). Many patients with sustained deep molecular response (DMR) can maintain remission after stopping tyrosine kinase inhibitors (TKIs), with a recent meta‐analysis of 2336 patients reporting TFR rates of 59% at 12 months and 55% at 24 months.^1^ However, most pivotal studies excluded patients with prior TKI resistance, including those with *BCR::ABL1* kinase‐domain mutations (KDMs), limiting applicability to less selected populations.

Data on TFR in patients with prior treatment failure remain scarce and heterogeneous across studies, although evidence supporting successful outcomes is increasing.^2^, ^3^, ^4^, ^5^, ^6^, ^7^, ^8^ Previously TFR guidelines have excluded patients who have not responded optimally. However, the recent 2025 European LeukemiaNet (ELN) guidelines^9^ state that TFR may be considered after resistance if durable DMR was achieved following a switch prompted by resistance due to a KDM sensitive to another TKI. We, therefore, evaluated TFR outcomes in CML‐CP patients with prior TKI failure who achieved stable DMR on subsequent therapy and assessed whether KDM influenced TFR probability. Our findings contribute to the discussion on expanding TFR eligibility in previously resistant patients.

Among patients with CML‐CP who discontinued TKI therapy at our institution, we retrospectively identified those with prior TKI failure due to resistance, defined by ELN 2025 criteria as an ‘unfavourable’ response at 3, 6 or 12 months or loss of MR^3^ (major molecular response [MMR], BCR::ABL1 ≤0.1% on the International Scale [IS]) while on therapy.^9^ Patients were then stratified according to the presence of a documented *BCR::ABL1* KDM. Inclusion criteria were receipt of TKI therapy for ≥3 years and sustained DMR for ≥1 year prior to TKI discontinuation. Patients without KDM analysis and those who restarted TKI before loss of MR^3^ were excluded. Only the first TKI discontinuation was considered. Outcomes of patients with a prior history of TKI failure were compared with those of a reference cohort (RC) of CML patients at our centre without such a history. The data cut‐off date was 30 November 2025.

Molecular monitoring was performed using *BCR::ABL1* ^IS^ and *BCR::ABL1* KDM analysis by Sanger sequencing, the standard‐of‐care method at our institution until October 2022. Molecular relapse was defined as confirmed loss of MR^3^ on two consecutive tests. TFR probability (pTFR) was estimated using the Kaplan–Meier method, and differences between groups were assessed using the log‐rank test. Patient were censored at their last molecular follow‐up while remaining in TFR. Continuous and categorical variables were compared using the Mann–Whitney *U*‐test and Fisher's exact test respectively. A two‐tailed *p* value <0.05 was considered statistically significant. The study was approved by our internal review board and all patients gave informed consent.

A total of 203 CML patients discontinued treatment and were included in the study: 59 patients interrupted TKI therapy after prior TKI failure (failure cohort, FC), of which 22 with documented *BCR::ABL1* KDM (KDM‐positive group, KDM^pos^g) and 37 without (KDM‐negative group, KDM^neg^g); 144 patients without TKI failure constituted the RC. Baseline characteristics at discontinuation are shown in Table 1, while *BCR::ABL1* mutations in the KDM^pos^g are provided in Table S1.

**TABLE 1:** Patient characteristics.

	Reference cohort (RC) (*n* = 144)	Failure cohort (FC)			*p* value (KDM^neg^g versus KDM^pos^g)
		All (*n* = 59)	KDM^neg^g (*n* = 37)	KDM^pos^g (*n* = 22)	
Females, *n* (%)	81 (56.3)	24 (40.7)	14 (37.8)	10 (45.4)	0.59
Median age at diagnosis, years (range)	46.2 (16–86)	48 (20–73.9)	46.4 (20–62.4)	50 (21.3–73.9)	0.22
Sokal					
Low/intermediate	70 (77.7)	30 (71.4)	21 (75)	9 (64.3)	0.49
High (missing)	20 (22.3) *54*	12 (28.6) *17*	7 (25) *9*	5 (35.7) *8*	
*BCR::ABL1* transcript type					
E14a2 (+e13a2)	90 (64.3)	35 (62.5)	19 (54.3)	16 (76.2)	0.15
E13a2	50 (35.7)	21 (37.5)	16 (45.7)	5 (23.8)	
Other/missing	*4*	*3*	*2*	*1*	
TKI first line					
Imatinib	114 (79.2)	58 (98.3)	37 (100)	21 (95.5)	0.37
≥2GTKI	30 (20.8)	1 (1.7)	0	1 (4.5)	
TKI at stopping					
Imatinib	75 (52.1)	8 (13.6)	6^a^ (16.2)	2^a^ (9.1)	0.7
≥2GTKI	69 (47.9)	51 (86.4)	31 (83.8)	20 (90.9)	
TKI line at stopping					
1st	95 (66)	8 (13.6)	6^a^ (16.2)	2^a^ (9.1)	0.7
≥2nd	49 (34)	51 (86.4)	31 (83.8)	20 (90.9)	
Number of TKIs failed					
1 (first line)	NA	48 (81.4)	34 (91.9)	14 (63.6)	*0.013*
≥2		11 (18.6)	3 (8.1)	8 (36.4)	
TKI dose at stop					
Lower than standard	123 (85.4)	51 (86.4)	31 (83.8)	20 (90.9)	0.7
Standard	21 (14.6)	8 (13.6)	6 (16.2)	2 (9.1)	
Response at stop					
MR^4^	19 (13.2)	20 (33.9)	17 (45.9)	3 (13.6)	*0.012*
≥MR^4.5^	125 (86.8)	39 (66.1)	20 (54.1)	19 (86.4)	
Median duration of TKI, years (range)	8.26 (3–21.6)	12.6 (3.1–22.2)	12.4 (5.2–22.2)	13.6 (3.1–21.5)	0.48
Type of TKI failure					
Primary	NA	38 (64.4)	26 (70.3)	12 (54.5)	0.27
Secondary		21 (35.6)	11 (29.7)	10 (45.5)	
Time to DMR, months (range)	19.6 (3.3–94.4)	54.9 (16.5–172.1)	71.1 (19.1–172.1)	47.2 (16.5–111.1)	0.056
Median duration of DMR, years (range)	6.2 (1–18.7)	5.7 (1–17.5)	5.4 (1–13.1)	6.6 (1.1–17.5)	0.22

Abbreviations: DMR, deep molecular response; KDM^neg^g, KDM‐negative group; KDM^pos^g, KDM‐positive group; TKI, tyrosine kinase inhibitors.

^a^ These patients overcame failure with high‐dose imatinib (600–800 mg daily) (in combination with homoharringtonine in 1 patient of the KDM^pos^g and with hydroxychloroquine in 1 patient in the KDM^neg^g) and achieved DMR on imatinib, maintained until TFR attempt.

All 59 patients failed at least their first‐line TKI, which was imatinib in the majority (*n* = 58). In the KDM^pos^g, patients had KDM either at the time of their first‐line TKI failure (*n* = 14), at both their first‐ and second‐line TKI failure (*n* = 1) or at second or later TKI‐line failure (*n* = 7).

All failure patients achieved DMR before TKI discontinuation, durable for a median of 5.4 years (1–13.1) and 6.6 years (1.1–17.5) in the KDM^neg^g and KDM^pos^g, respectively, and 54.1% and 86.4% of them were in MR^4.5^ or deeper response at stopping, respectively (*p* = 0.012). In addition to the depth of response, differences between the two groups were a higher proportion of patients failing only their first‐line therapy (91.9% vs. 63.6%, *p* = 0.013) and a longer time to first DMR (71.1 months vs. 47.2 months, *p* = 0.056) in the KDM^neg^g compared to the KDM^pos^g, respectively. Time to first DMR was much longer in the FC than in the RC, being 54.9 (16.5–172.1) and 19.6 months (3.3–94.4) (*p* < 0.0001), respectively.

The TKI line at interruption was ≥second line in most patients (90.9% and 83.8% in the KDM^pos^g and KDM^neg^g, respectively); however, eight patients were on first‐line imatinib. Of these, six in the KDM^neg^g and two in the KDM^pos^g had achieved sustained DMR after moving from standard dose (400 mg daily, SD) to high‐dose (600–800 mg daily, HD) imatinib alone (*n* = 6), in combination with homoharringtonine (*n* = 1, within a phase I/II academic study^10^) or with hydroxychloroquine (*n* = 1, within the CHOICES study^11^). In the two patients of the KDM^pos^g, mutations emerging while on SD imatinib were F317L and M244V, respectively, one each per patient. Ponatinib and asciminib were received only as the last TKI prior to discontinuation; four KDM^pos^g patients received ponatinib; and one KDM^neg^g patient received asciminib.

MR^3^ loss occurred in 57 (39.6%) and in 36 (61%) patients in the RC and in the FC, respectively. With the latter, 13 (59.1%) and 23 (62.2%) patients lost MR^3^ in the KDM^pos^g and in the KDM^neg^g, respectively.

The estimated 3‐year pTFR was 63.7% (95% confidence interval [CI]: 55.6–71.1) in the RC and 39.3% (95% CI: 27.5–52.5) in the FC (*p* = 0.002) (Figure 1A). Median time to MR^3^ loss was 3.7 (1–93.3) and 4 (1.1–83.8) months, respectively (*p* = 0.95). Median follow‐up of TFR patients was longer in the RC (79.8 months [6–199.5]) than in the FC (37.3 months [10.9–111.8]) (*p* = 0.001).

**FIGURE 1:**
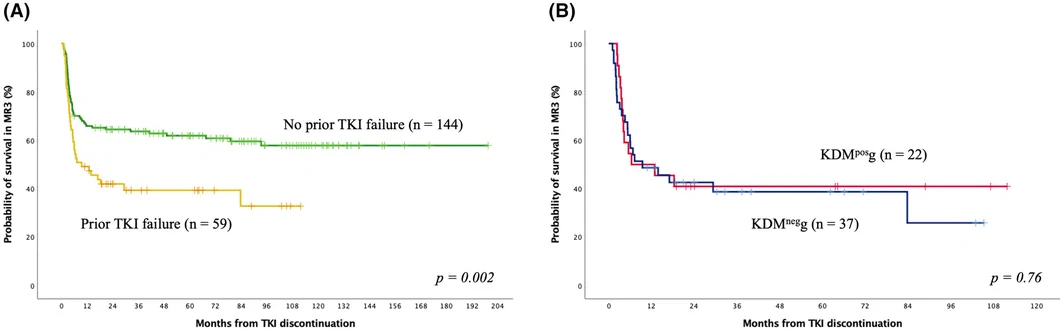
Treatment‐free remission (TFR) probability in chronic myeloid leukaemia (CML) patients stratified by (A) prior tyrosine kinase inhibitor (TKI) failure and (B) *BCR::ABL1* kinase domain mutation (KDM) status among those with prior TKI failure. (A) Kaplan–Meier curves comparing 3‐year TFR probability (pTFR) between the reference cohort (RC; *n* = 144 patients who attempted TFR without a history of TKI failure) and the failure cohort (FC; *n* = 59 patients with prior TKI failure due to resistance). The estimated 3‐year pTFR was 63.7% (95% CI: 55.6–71.1) in the RC and 39.3% (95% CI: 27.5–52.5) in the FC (*p* = 0.002). (B) Kaplan–Meier analysis restricted to the FC, stratified by *BCR::ABL1* KDM status: KDM‐negative and KDM‐positive (KDM^neg^g and KDM^pos^g, respectively). The 3‐year pTFR was 38.7% (95% CI: 24.4–55.3) in the KDM^neg^g and 40.9% (95% CI: 23.2–61.3) in the KDM^pos^g (*p* = 0.76).

Within the FC, the 3‐year pTFR was 38.7% (95% CI: 24.4–55.3) and 40.9% (95% CI: 23.2–61.3) in the KDM^neg^g and KDM^pos^g, respectively (*p* = 0.76) (Figure 1B). Median time to MR^3^ loss was 4.6 (1.1–83.8) and 4 (2.3–18.4) months, respectively (*p* = 0.72). Median follow‐up of TFR patients was longer in the KDM^pos^g (63.6 months [19–111.8]) than in the KDM^neg^g (35 months [10.9–105.3]), although the difference was not statistically significant (*p* = 0.44), likely due to low sample numbers (TFR patients in each group were 9 and 14 respectively).

Sixteen of 23 (69.6%) KDM^neg^g and seven of 13 (53.8%) KDM^pos^g patients resumed the same TKI after loss of MR^3^. Following TKI resumption, MR^3^ was regained by 22 of 23 (95.6%) and 12 of 13 (92.3%) patients, respectively, after a median of 2.8 (0.5–17) and 3.6 (0.5–27.9) months (*p* = 0.54; Tables S1 and S2). However, two patients in the KDM^neg^g discontinued dasatinib due to intolerance after re‐achieving MR^3^ but then developed Philadelphia‐negative secondary AML at 1.5 and 5 years from their MR^3^ loss and subsequently died. Both patients had a classical Philadelphia translocation, were high Sokal score and achieved sustained DMR for 6 and 4.2 years, respectively, before their first TKI discontinuation.

Of the two patients who failed to regain MR^3^, one in the KDM^neg^g had only 1.5 months of follow‐up after TKI resumption, whereas the other, in the KDM^pos^g, was treated with asciminib 20 mg once daily and only achieved MR^2^ after 27.5 months.

Eleven patients subsequently attempted TFR for the second time (TFR2) after sustaining DMR for at least 1 year. TFR2 was achieved by one of four (25%) KDM^pos^g and four of seven (57.1%) KDM^neg^g patients, who remained in DMR off TKI after a median follow‐up of 84.6 months (9–99).

Consistent with prior studies,^12^ we found that patients with prior TKI failure can reach TFR, although at a lower rate than optimal responders. Our observations also align with findings from Yuan et al.,^13^ who showed that TFR can be achieved in patients with prior TKI failure or accelerated‐phase disease, provided that sustained DMR is attained. Emerging evidence suggests that immunological mechanisms, particularly NK‐cell activity and adaptive immune surveillance, contribute to maintaining TFR once leukaemic burden is reduced.^14^

Among patients with prior TKI failure, we found no clear evidence that a history of KDM influenced TFR success. Three‐year pTFR rates were similar between mutation‐driven and mutation‐negative resistance. Furthermore, TFR2 in nearly half of FC patients attempting it reinforces the notion that TFR can remain a long‐term therapeutic goal for these patients.

This observation is relevant given the ELN 2025 recommendations,^9^ which restrict TFR beyond first‐line therapy to intolerance or mutation‐driven resistance sensitive to the alternative TKI.

Taken together, these results indicate that historical adverse features (prior TKI resistance, presence of KDM or initial accelerated‐phase disease) do not preclude TFR, provided sustained DMR is achieved. Of note, the median duration of DMR in the FC cohort substantially exceeded the minimum ELN 2025 criterion for attempting TFR, which may have contributed to the relatively favourable TFR rate.

Two KDM‐negative patients developed Philadelphia‐negative secondary AML. Such events are rare, with approximately 40 cases reported^15^ and a causal link with TKI discontinuation cannot be established.

Strengths of our study include the novel comparison of TKI failure by mutational status, the largest analysis to date of TFR after prior TKI mutation, extended follow‐up in the RC and the KDM^pos^g and single‐centre consistency.

Main limitations include its retrospective design, shorter follow‐up in the KDM^neg^g (although exceeded 3 years, when late relapses are uncommon) and small group sizes. Mutation analysis was performed using Sanger sequencing, which has lower sensitivity than next‐generation sequencing and may not detect low‐frequency subclonal mutations. However, Sanger reliably identifies dominant *BCR::ABL1* kinase domain mutations associated with overt TKI resistance, consistent with the focus of our study. Moreover, many patients currently considered for TFR were historically managed using Sanger‐based testing, supporting the relevance of our findings.

In conclusion, CML‐CP patients with prior TKI failure may be considered for TFR after achieving a durable DMR, regardless of whether the failure was mutation‐related. TFR in resistant patients should still be approached with caution, as TFR success rates remain lower than in optimal responders and two rare cases of Philadelphia‐negative secondary AML were observed.

These findings support an individualized, response‐driven approach to TFR eligibility and, if confirmed in larger studies, may inform future guideline updates.

## AUTHOR CONTRIBUTIONS

**Simone Claudiani:** Conceptualization; methodology; investigation; formal analysis; data curation; visualization; writing – original draft; writing – review and editing; project administration; software. **Silvia Metelli:** Writing – review and editing; supervision. **Myrto Katraki:** Methodology; data curation; writing – review and editing. **Fiona Fernando:** Writing – review and editing. **Andrew J. Innes:** Writing – review and editing. **Chloe Hayden:** Writing – review and editing; data curation; methodology. **Dragana Milojkovic:** Supervision; conceptualization; writing – review and editing; resources.

## FUNDING INFORMATION

The authors have nothing to report.

## CONFLICT OF INTEREST STATEMENT

S.C.: none; S.M.: none; M.K.: none; F.F.: none; A.J.I.: none; C.H.: none; D.M.: ELVN, TERNS, Ascentage, Novartis, Pfizer and Incyte (honoraria and research funding).

## DATE AVAILABILITY STATEMENT

The data analysed during the current study are available from the corresponding author upon reasonable request.

## ETHICS APPROVAL AND CONSENT STATEMENT

The study was approved by our internal review board and all patients gave informed consent.

## Supporting information


**Table S1.** *BCR::ABL1* kinase domain mutations identified, TKI line and outcome of TFR attempt for each CML patient in the KDM‐positive group.
**Table S2.** Management after MR^3^ loss in patients without KDM (KDM‐negative group).

## References

[1] Zheng Z , Tang H , Zhang X , Zheng L , Yin Z , Zhou J , et al. Treatment‐free remission after discontinuation of tyrosine kinase inhibitors in patients with chronic myeloid leukemia in the chronic phase: a systematic review and meta‐analysis. Discov Oncol. 2024;15(1):586. 10.1007/s12672-024-01444-9 PMC11499512 39441428

[2] Rea D , Nicolini FE , Tulliez M , Guilhot F , Guilhot J , Guerci‐Bresler A , et al. Discontinuation of dasatinib or nilotinib in chronic myeloid leukemia: interim analysis of the STOP 2G‐TKI study. Blood. 2017;129(7):846–854. 10.1182/blood-2016-09-742205 27932374

[3] Claudiani S , Apperley JF , Khan A , Khorashad J , Milojkovic D . Prolonged treatment‐free remission in chronic myeloid leukemia patients with previous BCR‐ABL1 kinase domain mutations. Haematologica. 2020;105(5):e225–e227. 10.3324/haematol.2019.234179 PMC7193492 31537692

[4] Shah NP , Garcia‐Gutierrez V , Jimenez‐Velasco A , Larson SM , Saussele S , Rea D , et al. Treatment‐free remission after dasatinib in patients with chronic myeloid leukaemia in chronic phase with deep molecular response: final 5‐year analysis of DASFREE. Br J Haematol. 2023;202(5):942–952. 10.1111/bjh.18883 PMC10524617 37246588

[5] Okada M , Imagawa J , Tanaka H , Nakamae H , Hino M , Murai K , et al. Final 3‐year results of the Dasatinib discontinuation trial in patients with chronic myeloid leukemia who received Dasatinib as a second‐line treatment. Clin Lymphoma Myeloma Leuk. 2018;18(5):353–360.e1. 10.1016/j.clml.2018.03.004 29610029

[6] Bonifacio M , Scaffidi L , Miggiano MC , Facchinelli D , Tosoni L , Pezone S , et al. A successful treatment‐free remission is achievable also by chronic myeloid leukemia patients lacking optimal requirements. Blood Cancer J. 2024;14(1):53. 10.1038/s41408-024-01025-7 PMC10965964 38531834

[7] Iurlo A , Cattaneo D , Consonni D , Castagnetti F , Miggiano MC , Binotto G , et al. Treatment discontinuation following low‐dose TKIs in 248 chronic myeloid leukemia patients: updated results from a campus CML real‐life study. Front Pharmacol. 2023;14:1154377. 10.3389/fphar.2023.1154377 PMC10076530 37033642

[8] Chen Y , Zhao H , Guo J , Zou J , He W , Han D , et al. Successful treatment discontinuation in CML patients with full‐dose and low‐dose TKI: results from real‐world practice. Front Pharmacol. 2023;14:1101743. 10.3389/fphar.2023.1101743 PMC9899816 36755944

[9] Apperley JF , Milojkovic D , Cross NCP , Hjorth‐Hansen H , Hochhaus A , Kantarjian H , et al. 2025 European LeukemiaNet recommendations for the management of chronic myeloid leukemia. Leukemia. 2025;39(8):1797–1813. 10.1038/s41375-025-02664-w PMC12310532 40646132

[10] Marin D , Kaeda JS , Andreasson C , Saunders SM , Bua M , Olavarria E , et al. Phase I/II trial of adding semisynthetic homoharringtonine in chronic myeloid leukemia patients who have achieved partial or complete cytogenetic response on imatinib. Cancer. 2005;103(9):1850–1855. 10.1002/cncr.20975 15786422

[11] Horne GA , Stobo J , Kelly C , Mukhopadhyay A , Latif AL , Dixon‐Hughes J , et al. A randomised phase II trial of hydroxychloroquine and imatinib versus imatinib alone for patients with chronic myeloid leukaemia in major cytogenetic response with residual disease. Leukemia. 2020;34(7):1775–1786. 10.1038/s41375-019-0700-9 PMC7224085 31925317

[12] Zambrotta GPM , Nicolini FE , Assouline S , Busque L , Pungolino E , Abruzzese E , et al. Risk of progression in chronic phase‐chronic myeloid leukemia patients eligible for tyrosine kinase inhibitor discontinuation: final analysis of the TFR‐PRO study. Am J Hematol. 2023;98(11):1762–1771. 10.1002/ajh.27073 37647134

[13] Yuan M , Zhang Y , Liu B , Zhou L , Zhu Y , Li Z , et al. Treatment‐free remission in chronic myeloid leukaemia patients with accelerated phase or tyrosine kinase inhibitor therapy failure. Br J Haematol. 2025;207(4):1408–1415. 10.1111/bjh.70080 PMC12512077 40799021

[14] Ilander M , Olsson‐Stromberg U , Schlums H , Guilhot J , Bruck O , Lahteenmaki H , et al. Increased proportion of mature NK cells is associated with successful imatinib discontinuation in chronic myeloid leukemia. Leukemia. 2017;31(5):1108–1116. 10.1038/leu.2016.360 PMC5420794 27890936

[15] Liu H , Sun Y , Li L , Zhang Y , Zhou Y , Zeng P , et al. Rapid development of Philadelphia chromosome‐negative AML in a CML patient with sustained major molecular response to tyrosine kinase inhibitor therapy. Leuk Res Rep. 2025;24:100536. 10.1016/j.lrr.2025.100536 PMC12409312 40917154

