# Supplementary material for: Treatment‐free remission in CML patients with prior TKI failure is not influenced by previous BCR::ABL1 kinase domain mutations
Source: Br J Haematol. 2026 Sep 2;209(5):2326–30. doi: 10.1111/bjh.70794 (PMC13748396; doi:10.1111/bjh.70794)
Supplement: Supplementary file 1 [file BJH-209-2326-s001.pdf]

Supplementary Table 1. *BCR::ABL1* kinase domain mutations identified, TKI line, and outcome of TFR attempt for each CML patient in the KDM-positive group.

UPN*	***BCR::ABL1* KD mutation**	**TKI line at the mutational test**	**TKI line at discontinuation**	**Outcome (TFR versus MR^3 loss)**	**Follow-up in TFR (months)**	**KD mutation at MR^3 loss**	**TKI after MR^3 loss**	**MR^3 re-achieved**	**DMR re-achieved**	**TFR2 attempt outcome**
2	L248V	2L Dasatinib	3L Nilotinib	MR^3 loss	-	L248V	Nilotinib	yes	yes	-
10	D276G	1L Imatinib	3L Dasatinib	MR^3 loss	-	NO	Dasatinib	yes	yes	-
14**	M244V	1L Imatinib	1L Imatinib	**TFR**	111.77	-	-	-	-	-
33	T315I	1L Imatinib	2L Ponatinib	**TFR**	88.83	-	-	-	-	-
82	D276G	1L Imatinib	2L Nilotinib	MR^3 loss	-	unknown	Dasatinib	yes	yes	Failed TFR2 attempt
84	M351T, H396R	1L Imatinib	3L Nilotinib	**TFR**	63.57	-	-	-	-	-
	F317L	2L Dasatinib								
88	T315I	2L Dasatinib	3L Ponatinib	**TFR**	107.13	-	-	-	-	-
89	L387M	1L Imatinib	3L Nilotinib	MR^3 loss	-	unknown	Bosutinib	yes	yes	-
99	V299L	2L Dasatinib	4L Nilotinib	**TFR**	19.03	-	-	-	-	-
102	T315I	3L Dasatinib	4L Ponatinib	MR^3 loss	-	unknown	Asciminib	yes	yes	-
110	H296R	1L Imatinib	2L Dasatinib	MR^3 loss	-	unknown	Dasatinib	yes	yes	In TFR2
206	Q252H	1L Imatinib	2L Nilotinib	**TFR**	64.23	-	-	-	-	-
208	V299L	1L Imatinib	2L Nilotinib	MR^3 loss	-	unknown	Nilotinib	yes	yes	-
213	K263E	2L Nilotinib	2L Nilotinib	MR^3 loss	-	unknown	Nilotinib	yes	yes	Failed TFR2 attempt
223	G250E, E255V	1L Imatinib	2L Dasatinib	MR^3 loss	-	unknown	Dasatinib	yes	yes	-
228	G250E	1L Nilotinib	2L Dasatinib	MR^3 loss	-	NO	Dasatinib	yes	yes	Failed TFR2 attempt
229	S417F	1L Imatinib	3L Nilotinib	MR^3 loss	-	NO	Bosutinib	yes	yes	-
289	F359V	2L Nilotinib	4L Ponatinib	MR^3 loss	-	K247R	Asciminib	no	no	-
	F317L	3L Dasatinib								
291**	F317L	1L Imatinib	1L Imatinib	**TFR**	24.10	-	-	-	-	-
318	F359C	2L Nilotinib	3L Dasatinib	**TFR**	23.03	-	-	-	-	-
320	E453V	1L Imatinib	2L Dasatinib	**TFR**	21.7	-	-	-	-	-
339	E279A	1L Imatinib	2L Dasatinib	MR^3 loss	-	NO	Bosutinib	yes	yes	-

* all patients but UPN 228 received imatinib as 1st line TKI and experienced treatment failure with it.
** these two patients received high-dose imatinib (600-800 mg daily) after the detection of the *BCR::ABL1* KD mutation, which was their TKI at stop; UPN 14 also received homoharringtonine in combination with imatinib.

Supplementary Table 2. Management after MR^3 loss in patients without KDM (KDM-negative group).

UPN	TKI line at discontinuation	TKI after MR^3 loss	MR^3 re-achieved	DMR re-achieved	TFR2 attempt outcome
30	2L Dasatinib	Dasatinib	yes	yes	In TFR2
36	2L Dasatinib	Nilotinib	yes	yes	Failed TFR2 attempt
55	3L Nilotinib	Nilotinib	yes	yes	-
73	1L Imatinib	Nilotinib	yes	yes	In TFR2
76	2L Dasatinib	Dasatinib	yes	yes	Failed TFR2 attempt
85	3L Dasatinib	Bosutinib	yes	no	-
92	3L Dasatinib	Dasatinib	yes	yes	-
98	2L Dasatinib	Dasatinib	yes	yes	In TFR2
105	2L Nilotinib	Bosutinib	yes	yes	-
107	1L Imatinib	Imatinib	yes	yes	Failed TFR2 attempt
113	3L Dasatinib	Dasatinib	yes	yes	-
184	2L Dasatinib	Bosutinib	yes	yes	In TFR2
191	1L Imatinib	Imatinib	yes	yes	-
205	2L Dasatinib	Dasatinib	no	no	-
219	2L Dasatinib	Dasatinib	yes	yes	-
259	3L Asciminib	Asciminib	yes	yes	-
261	2L Dasatinib	Nilotinib	yes	yes	-
263	2L Nilotinib	Nilotinib	yes	yes	-
287	4L Bosutinib	Bosutinib	yes	no	-
293	3L Nilotinib	Nilotinib	yes	yes	-
297	2L Dasatinib	Dasatinib	yes*	yes	-
314	2L Dasatinib	Dasatinib	yes	yes	-
325	2L Nilotinib	Asciminib	yes	yes	-

* UPN 297 regained MR^3 on dasatinib, then lost MR^3 and a T315I was detected. The patient switched to asciminib and died while in MR^3 from a non-CML cause.
